# miR548ai antagonism attenuates exosome-induced endothelial cell dysfunction

**DOI:** 10.1038/s41420-021-00720-9

**Published:** 2021-10-28

**Authors:** Xiujie Xie, Lian-Wang Guo, Craig K. Kent

**Affiliations:** 1 Department of Surgery, School of Medicine, University of Virginia, Charlottesville, VA 22908 USA; 2 Department of Molecular Physiology and Biological Physics, University of Virginia, Charlottesville, VA 22908 USA; 3 Robert M. Berne Cardiovascular Research Center, University of Virginia, Charlottesville, VA 22908 USA

**Keywords:** Genetics, Physiology

## Abstract

Endothelial cell (EC) and smooth muscle cell (SMC) are major cell types adjacent in the vascular wall. Recent progress indicates that their communication is crucial for vascular homeostasis and pathogenesis. In particular, dysfunctional (proliferative) SMCs through exosomes can induce EC dysfunction (impaired growth). The current study suggests that miR548ai, a rarely known microRNA, may provide a molecular target for protection against SMC/exosome-induced EC dysfunction. We performed microarray profiling of microRNAs of dysfunctional human primary aortic SMCs induced by different cytokines (PDGF-BB, TGFβ1, TNFα, IL1β). Among the microRNAs commonly upregulated by these cytokines, miR548ai showed the most robust changes, as also validated through quantitative PCR. This cytokine-induced miR548ai upregulation was recapitulated in the qPCR determination of SMC-derived exosomal microRNAs. Consistent with SMC-to-EC communication, the exosomes extracted from cytokine-stimulated SMCs impaired human EC proliferation and migration. Of particular interest, this SMC exosomal impingement on ECs was countered by transfection of miR548ai inhibitor microRNA into ECs. Furthermore, the miR548ai inhibitor transfected into SMCs attenuated SMC dysfunction/proliferation. Thus, these results identify miR548ai as a novel target; namely, miR548ai inhibitor mitigates EC dysfunction induced by exosomes derived from dysfunctional SMCs. This new knowledge may aid the future development of microRNA-based treatment of vascular disorders.

## Introduction

Vascular smooth muscle cell (SMC) and endothelial cell (EC) are two major cell types in the arterial wall. Adjacent to each other, they play distinct roles and their communication is essential for vascular homeostasis [1, 2]. Dyshomeostasis within and between these cells promotes vascular diseases, most prominently atherosclerosis—the leading cause of morbidity and mortality in developed countries [3]. Angioplasty is the most commonly used procedure to treat atherosclerosis by reopening occluded arteries. However, this paradoxically leads to restenosis, i.e. re-occlusion of treated arteries. Angioplasty damages ECs and SMCs exposing them to a myriad of stimuli, particularly cytokines. In response, ECs and SMCs undergo phenotypic changes and become dysfunctional due to the loss of their innate functions. Dysfunctional SMCs become proliferative and migratory amassing flow-obstructive neointima lesions, a process termed intimal hyperplasia (IH) that causes restenosis [4]. Dysfunctional ECs, on the other hand, manifest impaired growth and hence delayed re-sealing (re-endothelialization) of the angioplasty-denuded inner vessel wall. Evidence indicates that SMC dysfunction and EC dysfunction critically and synergistically contribute to IH [5]. While SMC dysfunction and EC dysfunction each has been intensively studied, their communication, especially the mechanisms by which dysfunctional SMCs adversely impact ECs, remain an under-investigated area [6, 7]. A better understanding of these mechanisms could lead to discoveries of novel molecular targets and ultimately, improved therapeutic methods.

Recently, exosomes were discovered to be a new cell–cell communication route, which as carriers of pathological information have attracted considerable attention [8–11]. Exosomes participate in a wide range of physiological and pathological processes by selectively packaging and transferring cargo (proteins, nucleic acids, lipids, and metabolites) to adjacent or long-distance recipient cells. In recent years, exosomes have received extensive interest for their important role in the development of cardiovascular disease [8, 12]. These membrane vesicles are enriched with diverse cargo molecules including a large number of miRNAs, which are a group of non-coding RNAs that can be taken up by recipient cells to fine-tune gene expression post-transcriptionally [13]. The expression and function of exosomal microRNAs are highly tissue-type and cell-type specific, and also pathophysiological context-dependent. Circulating exosomal microRNAs and those derived from mesenchymal stem cells, immune cells, and ECs have been extensively studied in the cardiovascular system [14–16], some recognized as novel biomarkers and/or potential therapeutics. However, the role of exosomal microRNAs derived from dysfunctional SMCs is under-explored, with reports sparsely emerging [6, 17, 18].

In this study, through unbiased microarray and bioinformatic analyses using human primary SMCs, we identified a group of microRNAs that were commonly upregulated in dysfunctional SMCs when induced with different cytokines. In particular, miR548ai which is little-known in the literature was most prominently upregulated. We then used exosomes extracted from cytokine-stimulated dysfunctional SMCs to induce EC dysfunction (impaired proliferation and migration). We found that this EC-detrimental effect of SMC-derived exosomes was mitigated by miR548ai’s inhibitor microRNA transfected into ECs. Moreover, SMC transfection with the miR548ai inhibitor attenuated SMC dysfunction. These findings together suggest that miR548ai antagonism may provide a new approach to prevent EC dysfunction that is induced by exosomes from dysfunctional SMCs.

## Results

### EC proliferation and migration are hampered by exosomes isolated from conditioned media of cytokine-activated aortic SMCs (AoSMCs)

**Fig. 1:**
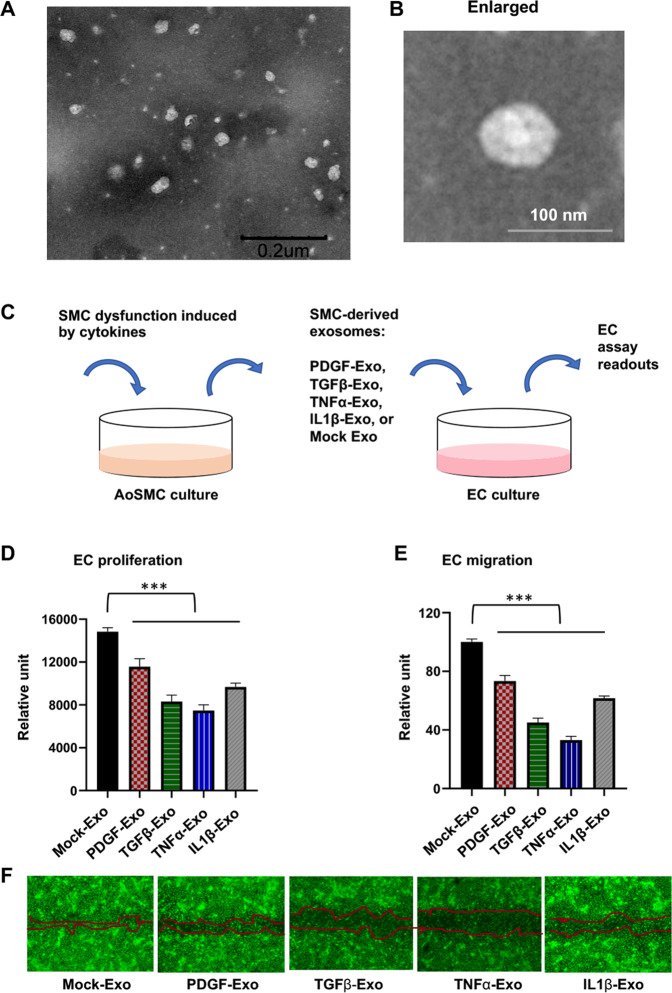
Inhibitory effect of dysfunctional SMC-derived exosomes on EC proliferation and migration. Human primary aortic smooth muscle cells (AoSMCs) were starved in basal medium (no FBS) and then treated for 24 h without (Mock control) or with a cytokine (50 ng/ml PDGF-BB, 20 ng/ml TGFβ1, 20 ng/ml TNFα, or 10 ng/ml IL1β). Exosomes extracted from a conditioned medium were added to human umbilical vascular ECs cultured in full medium and incubated for 48 or 24 h prior to measurements of proliferation and migration, respectively. Data are presented as mean ± SD (*n* = 3 replicates). ***P < 0.001 (compared to any of the cytokine conditions), as analyzed with one‐way ANOVA followed by Bonferroni post hoc test. **A** TEM image of SMC-derived exosomes. Purified exosomes were resuspended in PBS. Scale bar: 200 nm. **B** Enlarged image of a single exosome. Scale bar: 100 nm. **C** Schematic of the experimental design for this figure. **D** EC proliferation (Cell Titer-Glo assay). **E** EC migration (scratch assay). **F** Representative images showing cell-free gaps 24 h after scratch. Migration was measured as an increase in cell-reoccupied gap space 24 h (vs. 0 h) after scratching. Calcein was used to render cells fluorescent for imaging at the end of the 24 h experiment.

Recent progress points to a key role of exosomes in cell–cell communications [15, 18]. Exosomes are known to be enriched with microRNAs—regulatory factors that profoundly influence vascular cell and tissue functions [8]. To better understand how dysfunctional SMCs impact ECs, in this study, we first set out to investigate the role of exosomes secreted from human AoSMCs (see Fig. 1A). We used cytokines to render SMCs dysfunctional (proliferative/migratory), an approach established in others’ and our reports [6, 19–21]. After treating AoSMCs with PDGF-BB, TGFβ1, TNFα, or IL1β, exosomes were isolated from the respective medium and added to the EC culture. Transmission electron microscopy (TEM) illustrated round-shaped vesicles of ~30–100 nm diameter (Fig. 1A and B, in contrast to the negative control in Fig. S1), consistent with the morphology of purified exosomes [12, 22]. Interestingly, exosomes from each conditioned medium significantly inhibited EC proliferation and migration as compared to that from the control medium (Mock) with no cytokine added (Fig. 1C–F). These results suggest that exosomes from dysfunctional AoSMCs may carry some cargo factors that impair EC function.

### MicroRNA profiling identifies prominent up-regulation of SMC-derived miR548ai in cytokine-stimulated conditions

**Fig. 2:**
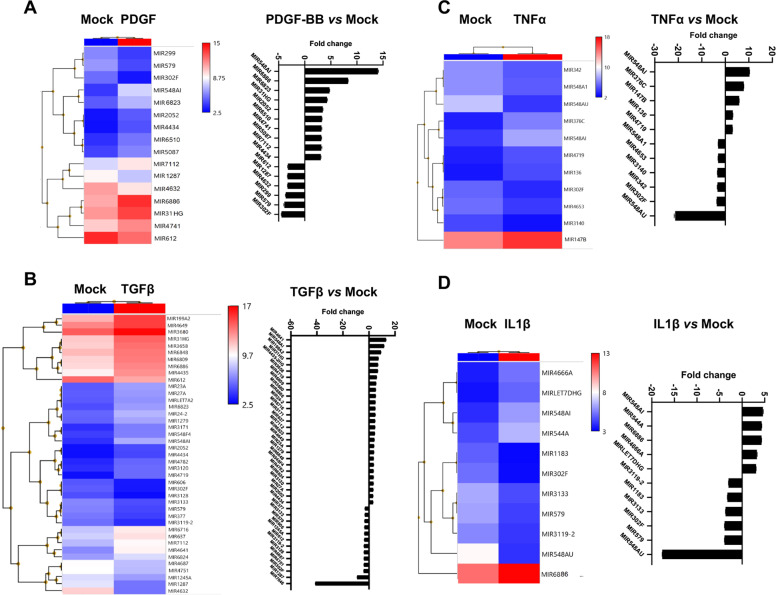
Profiles of SMC-derived, up- or down-regulated microRNAs after cytokine stimulation. Starved AoSMCs were cultured for 48 h without (Mock control) or with a cytokine (**A**: PDGF-BB, **B**: TGFβ1, **C**: TNFα, **D**: IL1β). Total RNA extracted from AoSMCs was used for microarray and microRNA profiling. Shown are heatmaps and fold-change profiles of microRNAs that meet the criterion of log2-fold change >2 (cytokine vs. Mock).

**Fig. 3:**
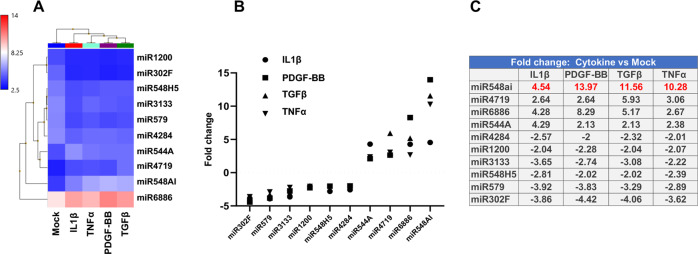
microRNAs that are commonly up- or down-regulated by all four cytokines. The AoSMC-derived microRNAs that were commonly up- or down-regulated by all four cytokines were selected (from the data in Fig. 2), and the data are presented as heatmap (**A**), plot (**B**), or table of fold changes (**C**).

We next performed microarray profiling of microRNAs. Starved AoSMCs were treated with either solvent control (Mock) or a cytokine (PDGF-BB, TGFβ1, TNFα, or IL1β) for 48 h. Total RNA was then prepared from each cell sample and used for microarray. Presented in Fig. 2 is the data selected with a filter of log2-fold change (>2 fold of increase or decrease). The heatmaps and respective plots indicate that stimulation of AoSMCs with different cytokines led to distinct profiles of up- and down-regulated microRNAs. However, by focusing on the microRNAs that were commonly up- or down-regulated in all four profiles, we found that miR548ai stood out as the most prominently up-regulated microRNA in all four cytokine-stimulated conditions (Fig. 3). The other microRNAs common in the four profiles included miR544A, miR4719, miR6886 (up-regulated), and miR302F, miR 579, temire, miR 1200, miR 548H5, and miR 4284 (down-regulated).

### qPCR validation confirms miR548ai upregulation in cytokine-stimulated AoSMCs and in exosomes thereof

**Fig. 4:**
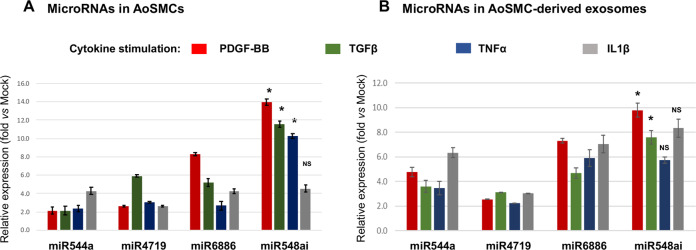
qRT-PCR determination of cytokine-stimulated upregulation of SMC-derived exosomal microRNAs. AoSMC culture, cytokine stimulation, and exosome isolation were performed as described in Fig. 1. qRT-PCR was performed to detect cytokine-induced level changes of the four microRNAs selected in Fig. 3. Levels of microRNAs in either cytokine-stimulated AoSMCs or AoSMC-derived exosomes were measured, as shown in **A** and **B**, respectively. The relative expression refers to microRNA fold change with (vs. without) cytokine stimulation. Data are presented as mean ± SD (*n* = 3 replicates). *P < 0.05, miR548ai compared to any of the other three microRNAs (between two bars of the same color), as analyzed with one‐way ANOVA followed by Bonferroni post hoc test; NS not significant.

To validate the profiling result of miR548ai, we performed TaqMan quantitative PCR (qPCR). Consistent with the microarray data, among SMC-produced microRNAs, miR548ai showed the most prominent upregulation (14 fold vs. Mock condition) relative to miR544A, miR4719, and miR6886, especially in the PDGF-stimulated condition (albeit not in the IL-1B condition) (Fig. 4A). In parallel, we also quantified these microRNAs in purified exosomes that were isolated from cytokine-stimulated AoSMCs. The qPCR results of microRNAs in exosomes (Fig. 4B) largely recapitulated that of microRNAs in AoSMCs (Fig. 4A). Specifically, exosomal miR548ai was most prominently upregulated in the PDGF-BB or TGFβ1-stimulated condition, although TNFα or IL-1β stimulated changes of miR548ai were not significantly greater than that of the other three microRNAs (Fig. 4B). Of particular interest, miR548ai has been scarcely studied overall and little explored in the vascular system. We, therefore, decided to focus on miR548ai in the following experiments to investigate its functional impact on ECs and SMCs.

### miR548ai inhibitor microRNA mitigates EC dysfunction induced by SMC-derived exosomes

**Fig. 5:**
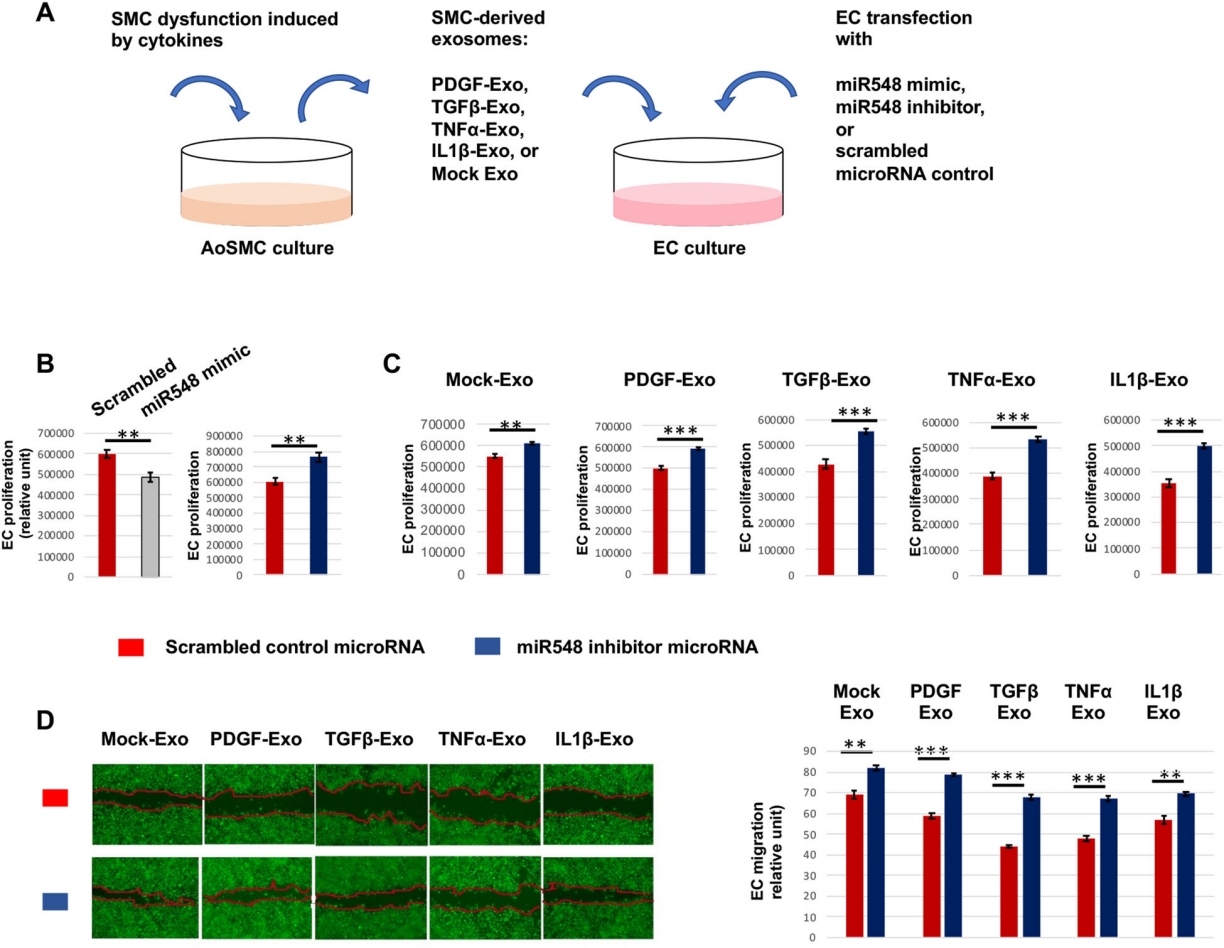
Effect of miR548ai inhibitor on EC proliferation and migration. **A** Schematic of the experimental design for this figure. **B** EC proliferation in full medium. ECs cultured in the full medium were transfected with a miR548ai mimic or inhibitor or scrambled microRNA (control) prior to Cell Titer-Glo assay. **C** and **D** EC proliferation and migration (respectively) in the presence of SMC-derived exosomes. Exosomes were isolated from the conditioned media of AoSMCs that were starved and then stimulated with a cytokine (same concentration as indicated in Fig. 1). The exosomes and miR548ai inhibitor (or scrambled microRNA) were added to ECs. Migration was measured as an increase in cell-reoccupied gap space 24 h (vs. 0 h) after scratching. Calcein was used to render cells fluorescent for imaging at the end of the 24 h experiment. Data are presented as mean ± SD (*n* = 3 replicates). Student *t*-test: **P < 0.01, ***P < 0.001.

To study the influence of miR548ai on EC growth, we transfected ECs with a miR548ai mimic or inhibitor microRNA (schematized in Fig. 5A). The data in Fig. 5B indicated that while miR548ai mimic hampered, miR548ai inhibitor promoted EC proliferation, consistent with its enhancing effect on levels of activated pro-growth gene products in ECs such as phosphorylated MEK/ERK and AKT (Fig. S2). This result and that from SMC-derived exosomes (Fig. 1) together suggested that miR548ai produced by cytokine-stimulated AoSMCs could have a paracrine effect on ECs, e.g. via exosomes. We thus transfected ECs with the miR548ai inhibitor and also treated ECs with exosomes derived from cytokine-stimulated AoSMCs to induce EC dysfunction. The purpose was to use miR548ai inhibitor to antagonize the function of miR548ai. Indeed, compared to scrambled microRNA control, miR548ai inhibitor markedly increased EC proliferation, either added with exosomes from cytokine-stimulated AoSMCs or from non-stimulated Mock condition (albeit to a minor extent) (Fig. 5C).

We then used the same experimental setting to determine EC migration, as shown in Fig. 5D. Similar to the result of EC proliferation, miR548ai inhibitor vs. scrambled microRNA control significantly increased EC migration in the presence of exosomes from cytokine-stimulated AoSMCs. Consistent with the functional specificity of miR548ai inhibitor, its effect on ECs was relatively minor in the presence of exosomes from non-stimulated AoSMCs where miR548ai production was lower. Taken together, these and the above results indicate that whereas exosomes derived from cytokine-activated AoSMCs impair EC proliferation and migration (Fig. 1), miR548ai inhibitor counters these adverse effects to bolster EC growth.

### miR548ai inhibitor mitigates cytokine-induced AoSMC dysfunction

**Fig. 6:**
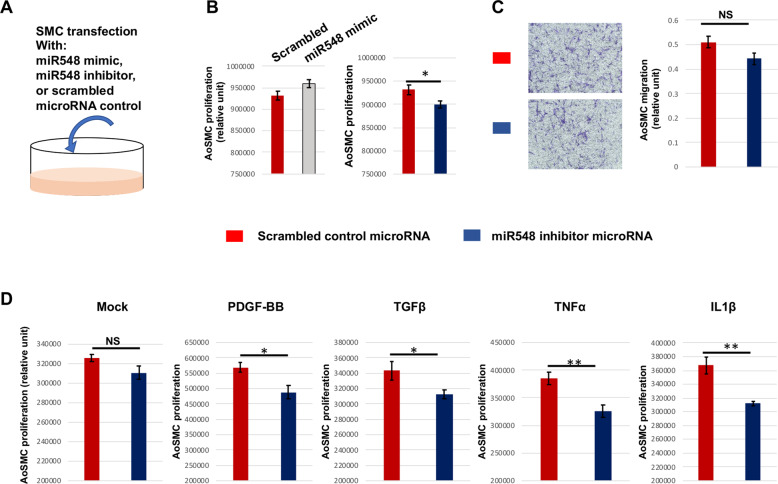
Effect of miR548ai inhibitor on AoSMC proliferation and migration. **A** Schematic of the experimental design for this figure. **B** SMC proliferation in full medium. AoSMCs cultured in the full medium were transfected with miR548ai mimic or inhibitor or scrambled microRNA (control) prior to CellTiter-Glo assay. **C** SMC migration in full medium. AoSMCs cultured in the full medium were transfected with miR548ai inhibitor or scrambled microRNA (control) prior to the Transwell migration assay. **D** SMC proliferation in defined medium. Starved AoSMCs were transfected with miR548ai inhibitor or scrambled control prior to stimulation with PDGF-BB, TGFβ1, TNFα, or IL1β (same concentrations as indicated in Fig. 1). Data are presented as mean ± SD (*n* = 3 replicates). Student *t*-test: *P < 0.05, **P < 0.01; NS not significant.

Contrary to ECs, proliferative SMCs can lead to adverse consequences such as the buildup of neointima lesions on the inner vascular wall that obstruct blood flow [19, 23]. We therefore next explored how miR548ai inhibitor influences SMC proliferation (diagramed in Fig. 6A). The data in Fig. 6B indicated that while transfection with the miR548ai mimic increased (*p* = 0.054), transfection with the miR548ai inhibitor microRNA reduced AoSMC proliferation in full-medium cultures. The miR548ai inhibitor also reduced AoSMC migration although the change did not reach statistical significance (Fig. 6C). This relatively low potency of miR548ai inhibitor could be attributed to a complex compendium of factors contained in the full medium (used in the migration assay) that may partially cancel the effect of miR548ai inhibitor. We then re-examined the effect of miR548ai inhibitor in a defined starvation medium with (or without) a cytokine added. The data in Fig. 6D showed that transfection with miR548ai inhibitor markedly attenuated AoSMC proliferation in the presence of cytokine stimulation. The effect of miR548ai inhibitor did not reach statistical significance in non-stimulated AoSMCs, likely due to the fact that relatively small amounts of miR548ai were produced in this Mock condition. In aggregate, our results indicate that transfection with miR548ai inhibitor not only improves EC growth but also mitigates SMC proliferation.

## Discussion

Emerging evidence suggests that while normal SMC-derived exosomes are crucial for vasculature homeostasis, dysfunctional SMC-derived exosomes contribute to vascular diseases [12, 17, 24]. In the current study, we searched for the microRNAs that could be targeted to counter EC dysfunction inflicted by exosomes from dysfunctional SMCs. We found that miR548ai was upregulated in SMC-derived exosomes in response to cytokine stimulation. More importantly, whereas dysfunctional SMC-derived exosomes impaired, miR548ai’s inhibitor microRNA boosted EC growth (proliferation/migration). Interestingly, the miR548ai inhibitor was also effective in attenuating SMC proliferation (dysfunction). While SMC proliferation and migration underlie the buildup of neointima lesion, EC dysfunction (impaired proliferation/migration, as opposed to SMCs) further exacerbates SMC dysfunction and IH [25]. Therefore, our results confer new information that implicates miR548ai as a potential interventional target for EC protection.

We used four different cytokines to induce SMC dysfunction and then performed unbiased screening through microRNA profiling to extract the information of common microRNAs that were responsive to all four cytokines. In normal conditions, the ECs that line the inner vessel wall keep SMCs insulated from adverse stimulants in the blood. However, upon the angioplasty procedure, this protective EC barrier is broken, and SMCs are abruptly exposed to various cytokines (typically PDGF-BB, TGFβ1, TNFα, and IL1β) which stimulate SMCs phenotypic changes and hence loss of its innate functions [26]. In this light, we envisioned that the use of multiple cytokines instead of only one represents a more robust experimental setting to mimic the complex in vivo microenvironment where dysfunctional SMCs reside.

To the best of our knowledge, it is a novel finding that miR548ai plays an important role in EC and SMC dysfunction. The miR548 family contains 69 human genes which are primate-specific and poorly conserved [27]. Not identified until a decade ago, this family has been scarcely investigated overall. While some family members, such as miR548-3p and miR548ar have been implicated as anti-oncogenic [28, 29], little is known about miR548ai, and its role in the vascular system has not been previously reported. Similar to miR548ai, miR6886 is little studied with no known vascular-associated function. As miR6886 was also upregulated in dysfunctional AoSMCs induced by cytokines, this information would lead to a new research direction to delineate its role in SMC and EC dysfunction.

Exosomes contain a variety of molecular cargos, including proteins, mRNAs, and microRNAs [13, 30–32]. Proteomics studies have indicated that exosomes mediate SMC-to-EC communication [24]. Exosomal microRNAs secreted from dysfunctional SMCs have been reported to contribute to coronary artery calcification [12, 17, 33]. However, exosomal microRNAs that mediate SMC-to-EC signal transmission in the context of IH are not well defined. Of note, a very recent report indicated a deficiency of miR1246, miR182, and miR486 in exosomes derived from pulmonary SMCs stimulated by PDGF-BB [6]. However, this may not inform exosomal microRNAs derived from aortic SMCs, as the pulmonary and systemic vascular systems represent distinct pathophysiological settings. Herein, we observed prominent up-regulation of miR548ai not only in AoSMCs but also in exosomes isolated from AoSMCs, as a result of cytokine stimulation of AoSMCs. Indicative of functional importance of miR548ai, while AoSMC-derived exosomes added to the EC culture promoted EC dysfunction, the ECs transfected with miR548ai inhibitor were resistant to this detrimental exosomal effect. This result could be interpreted with the antagonism of the miR548ai inhibitor against the miR548ai molecules either in AoSMC-derived exosomes that were added to ECs or miR548ai produced within ECs or both. Our data could not distinguish which was the major mechanism. Nevertheless, we detected a cytokine-stimulated increase of miR548ai in exosomes isolated from AoSMCs (Fig. 4B). Moreover, the effect of miR548ai inhibitor on mitigating EC dysfunction was overall stronger against cytokine-stimulated SMC-exosomes (containing more miR548ai) than that in the non-stimulated Mock condition (less exosomal miR548ai). Therefore, it would be a plausible speculation that SMC-derived exosomal miR548ai at least in part accounted for the observed exosomal impingement on ECs. Equally important, the miR548ai inhibitor protected against cytokine-induced EC dysfunction also in the absence of SMC-derived exosomes, implicating a potential of translational utility.

**Fig. 7:**
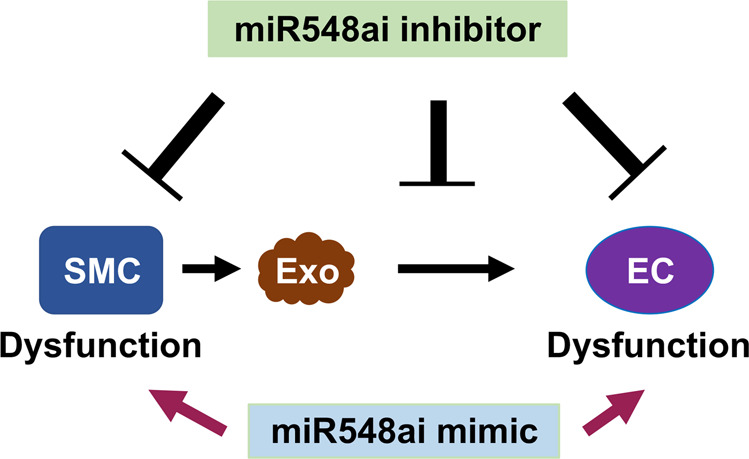
Schematic working model for the effect of miR548ai antagonism on EC and SMC dysfunction. Whereas exosomes (Exo) derived from dysfunctional SMCs induce EC dysfunction, miR548ai inhibitor mitigates this effect. miR548ai mimic exacerbates, and miR548ai inhibitor attenuates EC and SMC dysfunction.

We initially pursued microRNAs as potential targets for improving EC function. Serendipitously, our data showed that the use of miR548ai inhibitor not only met this purpose but also ameliorated SMC dysfunction (see schematic in Fig. 7). This dual effectiveness of targeting miR548ai is particularly interesting in view of the limitations of current therapeutic methods of targeting SMC dysfunction. Angioplasty is commonly performed to treat atherosclerosis [5]. This invasive procedure induces IH that re-narrows the vascular lumen, leading to restenosis [23]. While dysfunctional SMCs constitute the neointimal lesion, dysfunctional adjacent ECs are thrombogenic and they further promote SMC dysfunction and IH [25]. The current treatment for post-angioplasty IH is intraluminal implantation of a stent that releases an anti-proliferative drug to attenuate SMC dysfunction [34]. This method is inherently flawed because both the drug and the stent do not attenuate, but rather, exacerbate EC dysfunction, thereby heightening thrombogenicity while also exacerbating IH. It is therefore an urgent task for researchers to identify a common target to abrogate both SMC and EC dysfunction, thereby mitigating IH while protecting the endothelium. To this end, miR548ai provides a potential target for future translational development of endothelium-protective methods.

On the other hand, since little is known about miR-548ai’s expression and function across various human tissues and organs, systemic delivery of miR-548ai inhibitor is subject to off-target concerns, as observed with other microRNAs such as miR-21 inhibitor [35]. Indeed, while miR-548ai was proposed to be a biomarker significantly upregulated in bladder tumors based on data from urine exosomes [36], it was implicated to be anti-inflammatory and under-expressed in the amnion membranes of pregnant women with chorioamnionitis [37]. To mitigate off-target effects, an anti-miR-21-eluting stent has been used to implement *local* delivery to prevent experimental in-stent restenosis [35]. However, stent-free, lesion-targeted (e.g. to IH-prone arterial wall) endovascular microRNA delivery remains a challenge, in spite of an array of nanocarriers of microRNAs reported for intravenous injection [38].

## Conclusions

Cell–cell communication provides a refreshing perspective to interpret the innerworkings in health and disease, yet it is relatively less studied in the vascular field. The current manuscript reports a previously uncharacterized role of miR548ai, a rarely known member of the miR548 family. Specifically, whereas exosomes derived from dysfunctional SMCs impinge on EC growth, the SMC/exosome-induced EC dysfunction can be mitigated via EC transfection with a miR548ai inhibitor. Moreover, the application of miR548ai antagonism also attenuates SMC dysfunction. This multipotency of targeting miR548ai warrants further investigation into its potential translational significance in molecular intervention.

## Material and methods

### Materials

Human AoSMCs (CC-2571), human umbilical vein endothelial cells (HUVEC, CC-2517A), smooth muscle cell basal medium (SmBM-2, CC-3181), SmBM-2 plus SingleQuots of supplements (CC-3182), EBM-2 Basal Medium (CC-3156), and EGM-2 SingleQuots Supplements (CC-4176) were purchased from Lonza. Recombinant Human TGFβ1 (240-B), TNFα (210-TA), IL-1 beta (201-LB), and PDGF-BB (520-BB) were purchased from R&D Systems. Cell Titer-Glo 2.0 Assay kit was purchased from Promega (G9242). Transwell (12-mm diameter, 3.0 μm pore size) polycarbonate membrane insert was from Corning (3402). The following products were from Thermo Fisher Scientific: Total exosome isolation reagent (4478359), Total exosome RNA and protein isolation kit (4478545), scrambled microRNA control (AM4635), hsa-miR-548ai Mimic (Assay ID: MC22241), hsa-miR-548ai inhibitor (Assay ID: MH22241), Opti‐MEM I Reduced Serum Medium (31985062), Lipofectamine RNAiMAX Transfection Reagent (13778150), TaqMan MicroRNA reverse transcription kit (4366596), TaqMan Universal Master Mix II (4440043), TaqMan primers (hsa-miR548ai, Assay ID: 464169_mat; hsa-miR-544A, Assay ID: 002265; hsa-miR-4719, Assay ID: 464187_mat; hsa-miR-6886, Assay ID: 465086_mat; RNU44, Assay ID:001094).

### Human AoSMC and human umbilical vein endothelial cell cultures

AoSMCs and HUVECs were cultured in SmBM-2 or EGM-2 full medium (with supplements), respectively. Cells were passaged at a ratio of 1:4 when the confluence reached ~90% and used at passages 5–7. All cultured cells were maintained in a humidified incubator with 5% CO_2_ at 37 °C.

### Cytokine treatment of AoSMCs and exosome isolation from conditioned media

AoSMCs were cultured in the full medium until 90% confluence and starved in basal medium (no FBS) for 24 h. PDGF-BB (50 ng/ml), TGFβ1 (20 ng/ml), TNFα (20 ng/ml), or IL1β (10 ng/ml), was added to starved AoSMCs and incubated for 48 h. The conditioned media were collected and used for exosome isolation. We used the Total Exosome Isolation Reagent kit (ThermoFisher, cat. 4478359) according to the manufacturer’s recommendations. This kit was developed based on traditional methods with added benefits of good exosome yield and minimizing the need of tedious ultracentrifugation [22, 39]. In principle, the reagents force exosomes out of solution which can be collected via low-speed centrifugation [40]. Briefly, the collected conditioned medium was spun at 2000 × *g* for 30 min to remove cells and debris. The supernatant was mixed with Total Exosome Isolation Reagent (1/5 of medium volume) and incubated overnight at 4 °C. The precipitated exosomes were recovered at 4 °C by centrifugation at 10,000 × *g* for 60 min. Supernatants were aspirated and exosome pellets were re-suspended in 0.2 ml PBS and used immediately for experiments. Purified exosomes were quantified by using BCA Protein Assay Kit (ThermoFisher, cat.23225). Exosomes equivalent to 1 μg of proteins were used to treat ECs (10,000 cells). The presence of exosomes was confirmed via TEM in the Nanoscale Materials Characterization Facility at the University of Virginia. Briefly, the copper grid was washed with 0.01% BSA for 5 s. A 2 μl sample, either exosome in PBS or PBS alone (negative control) was loaded onto the grid. After 5 min, the grid was stained with 2% phosphotungstic acid for 1 min and dried. Images (40k) were taken using JEOL 1230 Transmission Electron Microscope.

### Microarray and microRNA profiling

AoSMCs were starved in basal medium (no FBS) for 24 h and then stimulated with PDGF-BB (50 ng/ml), TGFβ1 (20 ng/ml), TNFα (20 ng/ml), or IL1β (10 ng/ml) for 48 h. Total RNA was isolated (TRIzol reagent, ThermoFisher, cat. 15596026) from the cells and used for microarray, as we previously reported [19]. RNA integrity was interrogated using the Agilent 2100 Bioanalyzer (Agilent Technologies), and only the samples with an RNA integrity number >8 were used. Total RNA (100 ng per sample) was linearly amplified, and then labeled and fragmented using the GeneChip WT reagent kit (Affymetrix) according to the manufacturer’s instruction. Labeled cDNA fragments were hybridized to Affymetrix GeneChip human transcriptome Array 2.0 for analysis of gene expression. After wash and staining, the arrays were scanned using GeneChip Scanner 3000. Quality control, normalization, and comparison were analyzed in Affymetrix Expression Console. Gene-level differential expression analysis was performed using the Affymetrix Transcriptome Analysis Console software (version 4.0) using the paired-sample analytical pipeline. For default differential gene expression analysis, a log2-fold-change threshold of ±2 and an FDR of 10% were applied.

### Transfection of AoSMCs or ECs with miR548ai mimic or inhibitor

Cells were cultured in the full medium until 90% confluence and then in basal medium (0% fetal bovine serum) for 2 h. The cells were then transfected with hsa-miR548ai mimic, inhibitor, or scrambled control microRNA using Lipofectamine RNAiMAX Transfection Reagent (following the manufacturer’s instructions) for 12 h. Transfected cells were cultured in a fresh basal medium (no Lipofectamine) for another 12 h. PDGF-BB (50 ng/ml), TGFβ1 (20 ng/ml), TNFα (20 ng/ml), or IL1β (10 ng/ml) was then added, and cells were harvested for assays 24 h after cytokine treatment. For HUVEC transfection, cells were cultured in the full medium until 80% confluence and then in half basal/half-full medium (1% exosome-depleted fetal bovine serum) for 2 h. The cells were transfected with hsa-miR548ai mimic, inhibitor, or scrambled control microRNA using Lipofectamine RNAiMAX transfection reagent for 12 h and then cultured in fresh (no Lipofectamine) half basal/half-full medium for another 12 h. Exosomes purified from conditioned media were used to treat ECs, and the cells were harvested for assays 24 h after the treatment.

### TaqMan quantitative real‐time polymerase chain reaction (qRT-PCR)

Total RNA was extracted from cells using the TRIzol reagent following the manufacturer’s instructions. Total RNA from exosomes was extracted using Total Exosome RNA and Protein Isolation kit (ThermoFisher, cat. 4478545) following the manufacturer’s instructions. Isolated RNA was used for cDNA synthesis with the TaqMan MicroRNA reverse transcription kit (ThermoFisher, cat. 4366596). In each 20 μl reaction, 10 ng of cDNA was amplified through qRT‐PCR using TaqMan Universal Master Mix II (cat. 4440043), and mRNA expression was determined using a 7500 Real‐Time PCR System (Applied Biosystems). mRNA levels were normalized to RNU44 using the ∆∆Ct method. qRT‐PCR was performed in triplicate reactions.

### Cell Titer-Glo proliferation assay

Cells were washed once with PBS and then 50 μl of PBS plus 50 μl of CellTiter‐Glo reagent (per well) were added. Cell proliferation (viability) was analyzed using BioTek Gene 5 Microplate Reader (BioTek Instruments, Inc) to read 96‐well plates.

### Scratch and transwell migration assays

ECs were grown in 12-well culture plates to 90% confluence. After starvation for 24 h, cells (with/without transfection) were scratched with a sterile pipette tip across the cell monolayer followed by treatment of with exosomes derived from cytokine-stimulated AoSMCs for 20 h. For quantification of migration, the scratched area that was re-occupied by migrating cells after 20 h of treatment was determined using a microscope. The Transwell method was used for AoSMCs. Cells were grown in the Transwell insert until 90–95% confluence and then starved for 24 h in basal medium prior to microRNA transfection. The culture was changed to full medium in the lower chamber of Transwell. The inserts were washed three times with PBS after 24 h migration. The cells on the inside of the Transwell were removed using cotton swabs. The cells on the lower surface of the membrane were stained with 0.1% crystal violet (10% methanol) for 20 min. The membrane was washed three times with PBS to remove unbound crystal violet and then air-dried. The stained migrated cells were imaged under a microscope. After microscopy, 400 μl of 33% (v/v) acetic acid was added into the insert to elute the bound crystal violet by shaking for 10 min. The eluent collected in the lower chamber was measured for absorbance (590 nm) using a 96-well microplate reader.

### Statistical analysis

Prism 8.0 (GraphPad Software) was used for statistics. The sample size was determined through power analysis. Differences between the two groups were analyzed by Student’s *t*-test. For multi-condition comparison, we applied one‐way analysis of variance (ANOVA) followed by the Bonferroni post hoc test. *P* < 0.05 was considered statistically significant. Significance in all figures is indicated as follows: *P < 0.05, **P < 0.01, ***P < 0.001.

## Supplementary information

supplemetal

## Data Availability

Original microarray data has been deposited (GEO accession number: GSE185784).
