# Correction: miR548ai antagonism attenuates exosome-induced endothelial cell dysfunction

**DOI:** 10.1038/s41420-022-01178-z

**Published:** 2022-09-12

**Authors:** Xiujie Xie, Lian-Wang Guo, Craig K. Kent

**Affiliations:** 1 Department of Surgery, School of Medicine, University of Virginia, Charlottesville, VA 22908 USA; 2 Department of Molecular Physiology and Biological Physics, University of Virginia, Charlottesville, VA 22908 USA; 3 Robert M. Berne Cardiovascular Research Center, University of Virginia, Charlottesville, VA 22908 USA

**Keywords:** Genetics, Physiology

Correction to: *Cell Death Discovery* 10.1038/s41420-021-00720-9, published online 28 October 2021

The original version of this article contained an error in an author name. The name of the corresponding author Dr. Kent should be “Craig K. Kent”, currently Craig is listed as the middle name. We apologize for the error. The original article has been corrected.

