# Supplementary material for: miR548ai antagonism attenuates exosome-induced endothelial cell dysfunction
Source: Cell Death Discov. 2021 Oct 28;7:318. doi: 10.1038/s41420-021-00720-9 (PMC8553949; doi:10.1038/s41420-021-00720-9)
Supplement: Supplementary file 1 — supplemetal [file 41420_2021_720_MOESM1_ESM.docx]

**miR548ai antagonism attenuates exosome-induced endothelial cell dysfunction**

Xiujie Xie, PhD^1^; Lian-Wang Guo^1,2,3,*^, PhD; K. Craig Kent^1,*^, MD

^1^Department of Surgery, School of Medicine, University of Virginia, Charlottesville, VA 22908, USA

^2^Department of Molecular Physiology and Biological Physics, University of Virginia, Charlottesville, Virginia 22908, United States.

^3^Robert M. Berne Cardiovascular Research Center, University of Virginia, Charlottesville, VA 22908, USA

*Running title: miR548ai as a new target*

* Corresponding authors:

Lian-Wang Guo, Ph.D.

Department of Surgery, School of Medicine, University of Virginia, 409 Lane Road, Charlottesville, VA 22908, USA

Tel: +1 614 292 5276. Email: lg8zr@virginia.edu

K. Craig Kent, M.D.

1415 Jefferson Park Avenue #4027, Charlottesville, VA 22908, USA. Tel: 434-924-1082.

CK8AQ@hscmail.mcc.virginia.edu.

**Supplemental Figures**

**
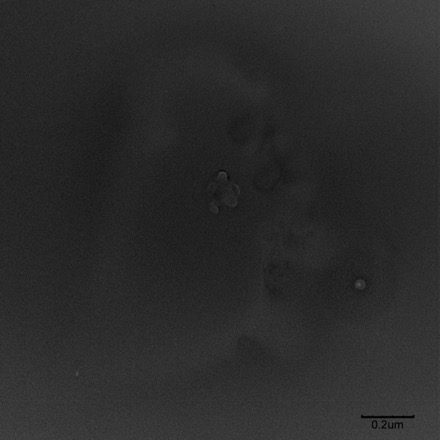
**

**Figure S1. Negative control of TEM imaging of exosomes**

Exosomes purified from human primary aortic smooth muscle cells (AoSMCs) were resuspended in PBS and subjected to TEM (see Figure 1A). The PBS buffer was used for negative control. Scale bar: 200 nm.

**Figure S2. Possible miR548ai’s target gene products in ECs**

ECs (HUVEC) cultured in full medium were transfected with miR548ai inhibitor or scrambled control (SCR), the cells were then used for Western blot analysis of proteins.
